# Group 2 Innate Lymphoid Cells Are Involved in Skewed Type 2 Immunity of Gastric Diseases Induced by *Helicobacter pylori* Infection

**DOI:** 10.1155/2017/4927964

**Published:** 2017-09-12

**Authors:** Rong Li, Xiao-xia Jiang, Lin-fang Zhang, Xiao-ming Liu, Ting-zi Hu, Xiu-juan Xia, Ming Li, Can-xia Xu

**Affiliations:** ^1^Department of Gastroenterology, Third Xiangya Hospital of Central South University, Changsha, China; ^2^Hunan Key Laboratory of Nonresolving Inflammation and Cancer, Changsha, China; ^3^Department of Gastroenterology, The Central Hospital of Yongzhou, Yongzhou, China

## Abstract

*H. pylori* induces a complicated local and systematic immune response and contributes to the carcinogenesis of gastric cancer. A primary type 1 immune response is evoked by *H. pylori* since its occurrence. However, it is not unusual that an inhibitory immunity is dominant in *H. pylori*-associated diseases, which are promoted by the formation of immunosuppressive microenvironment. But whether group 2 innate lymphoid cells (ILC2s) plays a critical role in *H. pylori*-induced skewed type 2 immunity is still unclear. In the present study, firstly, we confirmed that type 1 immunity was inhibited and type 2 immunity were undisturbed or promoted after *H. pylori* infection in vitro and in vivo. Secondly, GATA-3 was firstly found to be increased in the interstitial lymphocytes from *H. pylori*-associated gastric cancer, among them, Lin−GATA-3^+^ cells and Lin^+^GATA-3^+^ cells were also found to be enhanced, which indicated an important role for ILC2s in *H. pylori* infection. More importantly, ILC2s were found to be increased after *H. pylori* infection in clinical patients and animal models. In conclusion, our results indicated that ILC2-mediated innate immune response might play a potential role in dominant type 2 phenotype and immunosuppressive microenvironment in *H. pylori* infection.

## 1. Introduction

*Helicobacter pylori* (*H. pylori*) is a small, curved gram-negative bacterium with high motility; it colonizes the epithelium of human beings' stomach. According to epidemiological surveys, *H. pylori* has infected half of the world's population and has been proved to be the type I causative agent of gastric adenocarcinoma [1], which is the second most common diagnosed cancer and second most common cause of cancer-related deaths worldwide [2]. Gastric cancer is commonly classified into two subtypes based on its histological architecture, including the intestinal type and the diffuse type. Both subtypes have been tightly associated with *H. pylori*, and the chronic inflammation elicited by *H. pylori* very likely offers the setting in which gastric cancer initiation can occur [3]. Even so, only few individuals with *H. pylori*^+^ population experience a discomfort of the stomach caused by its colonization, and there are nearly 1-2% of infected individuals developing gastric adenocarcinoma eventually. However, no single universal explanation has yet been found to hold true across all populations studied, and it is likely that a combination of multiple factors determines the infection-related outcome.

The immune system including cellular immunity and humoral immunity will be activated when the defense barrier is destroyed by microorganisms. In most cases, the type of specific immunity induced is of key importance for protection. However, under certain circumstances, an improper response can even promote the induction of immunopathology [4, 5]. As a common sponsor for chronic gastric inflammation, persistent survival of *H. pylori* is always accompanied with activated local and systemic immune response, which has been derived from results of plenty of independent studies [6, 7]. Inflammation with *H. pylori* infection has widely been suggested to trigger gastric carcinogenesis through “inflammation-carcinoma chain.” *H. pylori* possesses an important ability on recruitment and differentiation of lymphoid cells in the stomach.

Neutrophils, macrophages, dendritic cells, and T cells are usually effector cells caused by *H. pylori* infection. Neutrophils are suggested to contribute to the clearance of *H. pylori* at least partly by recruiting more effective cells to the gastric mucosal epithelium [3]. T cells play a crucial role in the outcome of *H. pylori* involvement [8]. Most T cells in the resting state, named as Th0, show neither polarized phenotype but hold the potency to differentiate into type 1 or type 2 effective T helper cells (Th1 or Th2), which represent a predominant type 1 or type 2 cytokines, respectively. Both bacterial virulence and host genetic factors fluctuate the Th1 or Th2 differentiation mainly by promoting the leader cytokines in the microenvironment. IL-12, IL-18, and IFNs are powerful stimuli for Th1 development, whereas IL-4 promotes Th2.

During the development of *H. pylori* infection-associated pathologies, the levels of IL-12 and IL-2 gradually decreased, but IL-10 increased, which indicated some transformation in the patterns of cellular to humoral immunity. It has been demonstrated that a polarized type 2 immunity, but not type 1 immune response, is more frequently found in the chronic gastritis, precancerous lesions, and gastric adenocarcinoma caused by *H. pylori* infection [9]. An increasing tendency of IL-4 expression accompanies with the progression of gastric lesions, leading to a gradual decrease in Th1/Th2 ratio in CagA^+^* H. pylori*-infected patients [9, 10]. Moreover, it indicates that *H. pylori* stimulates a type 2 inflammatory response, which constitutes the significant character of its immunopathogenesis. These not only result in persistent survival of the bacterial pathogen that initiates the carcinoma but also promote a microenvironment for immune escape.

The highly complex host responses are involved in the coordination of innate and adaptive immune cell types, and the immunity to *H. pylori* infection is not exceptional. Not the adaptive immune cells, but the innate immune cells, as the important part of defending the barrier, react promptly to the alien invader at an early stage, and they evoke a timely defense. Innate lymphoid cells (ILCs), newly identified lymphocyte subsets, have thus attracted interests of many researchers in the fields of infectious disease [11]. ILCs are distributed in a wide variety of epithelial compartments and act as an intermediate position between acquired immune cells and bone marrow cells. Based on their cytokine production patterns that correspond to the T helper cell subsets Th1, Th2, and Th17, ILCs are commonly classified into three groups: ILC1, ILC2, and ILC3, respectively [11].

GATA-3, a critical regulator for type 2 immunities including ILC2s and Th2 cells, has been firstly found upregulated in *H. pylori*-associated gastric cancer from our previous differential screening of transcription factor expression [12]. The zinc-finger transcription factor, GATA-3, has received much attention as a master regulator of Th2 cell differentiation. More recently, apart from Th2 cells, ILC2, an important member of innate immunity, has been demonstrated to be another vital source of type 2 cytokines. What is more important is that GATA-3 was shown to contribute to type 2 immunity through regulation of ILC2 development and function [13, 14]. ILC2s potentiate and synergize memory Th2 cell responses in many ways [15, 16]. Moreover, it has been demonstrated that ILC2s were necessary for Th2 cell differentiation and maintenance [17, 18]. Therefore, a potential role of ILC2s in dominant type 2 immunity caused by *H. pylori* infection has attracted our interest.

In this study, by using clinical samples (plasmas and PBMCs from healthy volunteers (normal), individuals with chronic atrophic gastritis (CAG), and individuals with gastric adenocarcinoma (GC)) and establishing an *H. pylori* infection model *in vivo* and *in vitro*, we have demonstrated that immune response showed a gradual predominant trend for type 2 immunity in the progression of *H. pylori*-induced pathologies, when compared to a decreasing trend for Th1. GATA-3 expression has been firstly found to increase in lymphocytes derived from *H. pylori*^+^ gastric cancerous tissues. An experimental model of *H. pylori* infection established *in vitro* proved that *H. pylori* infection induced a leading type 2 immune response by enhancing the transcription factor GATA-3 and an increased population of Lin^−^GATA-3^+^. The latter indicated a vital phenotype for ILC2s. Moreover, the lymphocytes from clinical patients and an *H. pylori*-infected animal model have revealed that an incremental ILC2s might play a key role in the immune response to *H. pylori* infection.

## 2. Materials and Methods

### 2.1. Clinical Tissue Preparation

Clinical specimens were collected from 120 patients and healthy volunteers who underwent gastroscopy screening and diagnosis at the Third Xiangya Hospital of Central South University between May 2015 and February 2017. All informed consents were acquired from patients before sampling. To conduct cytokine chip hybridization and further detection, specimens and blood samples from *H. pylori*-positive^+^ or *H. pylori*-negative (−) chronic atrophic gastritis (CAG) and gastric cancer (GC) individuals were collected; moreover, normal samples offered by healthy volunteers were used as the control.

### 2.2. ELISA (Enzyme-Linked Immunosorbent Assay) for Cytokines

Plasma levels of IL-4, IL-5, IL-13, and IFN-*γ* (Shanghai ExCell Biology, China) were measured by the ELISA kit following the manufacturer's protocols. Concentration was calculated according to the standard curve.

### 2.3. PBMC (Peripheral Blood Mononuclear Cell) Isolation

Heparinized peripheral blood diluted with 1 : 1 phosphate-buffered saline (PBS) was layered over an equal volume of Ficoll and centrifuged as per the manufacturer's instructions. Mononuclear cells were collected from the interface between Ficoll and plasma. The collected PBMCs were washed by PBS for 3 times for the next intervention.

### 2.4. Quantitative Real-Time PCR (qRT-PCR)

Expressions of IL-4, IL-5, IL-13, IFN-*γ*, and GATA-3 genes were analyzed by qRT-PCR. Briefly, total RNA was extracted, respectively, from the above-mentioned samples by using the RNeasy Mini kit, and total RNA was reversely transcribed into cDNA using random primers and M-MLV reverse transcriptase (Qiagen) according to the manufacturer's recommendations. RT-PCR was built by using the iScript™ two-step RT-PCR kit with SYBR Green (Invitrogen) and the targeting gene primers. All gene primer sequences are shown in Table 1. PCR was performed in triplicate on the real-time PCR detection system with the following cycling parameters: 95°C (1 min), 40 cycles of 95°C (15 s), and 60°C (60 s). The qRT-PCR data were quantified by 2^−△△Ct^.

### 2.5. Immunohistochemistry Staining (IHC)

The rapid PV two-step staining method with the following specifications was used: paraffin slice thickness is 4 *μ*m; slices were grilled at 65°C for 60 min and then dewaxed in xylene and rehydrated in an increasing diluted ethanol series; high-temperature antigen retrieval was performed via a microwave in 0.1 M citrate solution (pH 6.0) for 10 min; slices were incubated with 3% H_2_O_2_ at room temperature for 20 min, with goat serum at room temperature for 20 min, and with anti-GATA-3 rabbit polyclonal antibody (Cell Signaling Technology, USA; diluted 1 : 200) at 4°C overnight; slices were rewarmed in the next day and then incubated with the second anti-rabbit antibody at room temperature for 20 min, after being washed with PBS; DAB coloration was performed; hematoxylin were mounted; and microscopic examination was conducted.

### 2.6. Tumor-Infiltrating Lymphocyte Preparation

Tumor tissues were taken from the center of the cancer immediately after surgical removal and histologic confirmation. For preparation of single cell suspensions, tumor samples were mechanically dissected and incubated with 1% type IV collagenase supplemented with 100 U/ml penicillin and 100 mg/ml streptomycin at 37°C for 30 minutes. The cell suspension was passed through a sterile 40 *μ*m nylon filter (BD Falcon, Heidelberg, Germany) and then was layered over an equal volume of Ficoll and centrifuged as per the manufacturer's instructions. Mononuclear cells were collected and suspended in PBS/culture medium.

### 2.7. Cell Culture and Coculture System

The human gastric epithelial cell GES-1 or freshly isolated PBMCs were grown in RPMI-1640 (Gibco/Life Technologies, Grand Island, NY) plus medium supplemented with 10% (vol/vol) heat-inactivated fetal bovine serum (Sijiqing, China), 100 U/ml penicillin, and 100 mg/ml streptomycin in a controlled humidified atmosphere in an incubator containing 5% CO_2_ and were passaged with 0.25% trypsin when necessary.

A transwell chamber (pore size = 3 *μ*m; Corning Inc.) was used for the coculture system. 1 × 10^4^ GES-1 was planted on the upper chamber of cell culture inserts overnight; then, live *H. pylori* or *H. pylori* lysates (ultrasonic crushing of *H. pylori*) were added into the upper chambers and 1 × 10^6^ freshly isolated PBMCs were separately seeded into the lower culture plates; these cells were cocultured. After coculture for 24 hours, PBMCs, GES-1, and supernatants from the lower chamber were collected for further analysis.

### 2.8. Bacterial Culture

The *H. pylori* strains SS1 (Western type) and ATCC 43504 were donated by Professor Yong Xie (Department of Gastroenterology, First Affiliated Hospital of Nanchang University, Jiangxi, China) and maintained in our laboratory. East Asian-type CagA^+^* H. pylori* and CagA^−^* H. pylori* were isolated from gastric ulcer patients' specimens during gastroscopy, respectively. They were grown on a Columbia blood agar plate supplemented with antibiotics (10 mg/l vancomycin, 5 mg/l cefsulodin, 5 mg/l amphotericin, and 5 mg/l trimethoprim) and 10% sheep blood (Bianzhen Biotech, Nanjing, China) at 37°C in microaerophilic conditions (5% O_2_, 10% CO_2_, and 85% N_2_) for 3-4 days. Then, the *H. pylori*, which was in the early log phase with good motility and activity for subculture or intervention, was harvested and resuspended in PBS. *H. pylori* concentration was estimated by measuring the OD_600 nm_, where OD_600 nm_ corresponds to about 2 × 10^8^ CFU (colony-forming unit)/ml. *H. pylori* was added to GES-1 at a MOI (multiplicity of infection) = 100, 200, or 400.

### 2.9. Animal Model

Twenty 4–6-week-old male C57BL/6 mice were purchased from the Department of Laboratory Animal, Third Xiangya Hospital of Central South University (Changsha, China); they were allocated into 4 groups randomly: the East Asian group (E), Western group (W), CagA^−^ group (C), and normal control group (N). After fasting for 24 hours, these mice were inoculated with East Asian-type CagA^+^* H. pylori*, Western-type CagA^+^* H. pylori*, and CagA^−^* H. pylori* by intragastric gavages (once every two days and lasted for a week), respectively. The normal controls were carried out with saline gavages. All mice were dieted with the same food and sterile water. The mice were sacrificed 6 weeks after intragastric gavages. Rapid urease test (RUT) and pathological biopsy were employed for detecting *H. pylori* infection. Splenocytes were obtained for preparation of lymphocyte suspensions. The gastric antrum tissues were collected and maintained in liquid nitrogen immediately for further histological detection and gastric tissue homogenate preparation. Medical ethics protocol was approved by the IRB of the Third Xiangya Hospital of Central South University.

### 2.10. Flow Cytometry Analysis

After the cleavage of red blood cells by Red Blood Cell Lysis Buffer (BD Pharmingen™, San Jose, CA, USA), lymphocyte suspensions from splenocytes were obtained for further analysis. For GATA-3 analysis, PBMCs, TILs, or splenocytes were collected and washed in PBS; then, the cells were fixed, permeabilized, and stained at 4°C with PE-conjugated GATA-3 as above before analysis. For ILC2 analysis, cells were labelled with antibodies to lineage markers (FITC-conjugated CD3, CD14, CD16, CD19, CD56, and Fc*ε*RIa) and antibodies to CD127 and CRTH2 (BD Pharmingen, San Diego, CA, USA) for 30 min at 4°C. Isotype control antibodies were used to determine positive cells. After staining, cells were washed or fixed with 1% paraformaldehyde and analyzed by using a FACS Canto II system (BD, Franklin Lakes, NJ, USA).

### 2.11. Statistical Analyses

Data were presented as the means ± standard deviations. Statistics were performed by using paired and unpaired Student *t*-tests as appropriate for each set of experimental conditions. *p* < 0.05 was considered statistically significant.

## 3. Result

### 3.1. A Skewed Type 2 Immune Response Was Found in the Progression of *H. pylori* Infection-Induced Gastric Pathologies

Types of strains, host gene polymorphism, and local immune microenvironment are involved in the outcome of the disease. *H. pylori* has been known to induce a particular local and systemic immunity. In order to clarify the dynamic regulation of immune response induced by *H. pylori*, an overview of cytokines was conducted, and we found a discrepant cytokine profile in the *H. pylori*^+^ chronic atrophic gastritis (CAG) or gastric cancer (GC) individuals, when compared to the healthy volunteers (NGM) (unpublished data). IFN-*γ*, representative of type 1 immune response, decreased in CAG and GC. On the contrary, the type 2 cytokines, IL-4 and IL-5, were stimulated in CAG and GC. In other words, an inhibited type 1 immunity and a relatively prevailed type 2 immunity were found in the microenvironments of *H. pylori*^+^ persistent chronic atrophic gastritis and gastric carcinoma.

**Figure 1:**
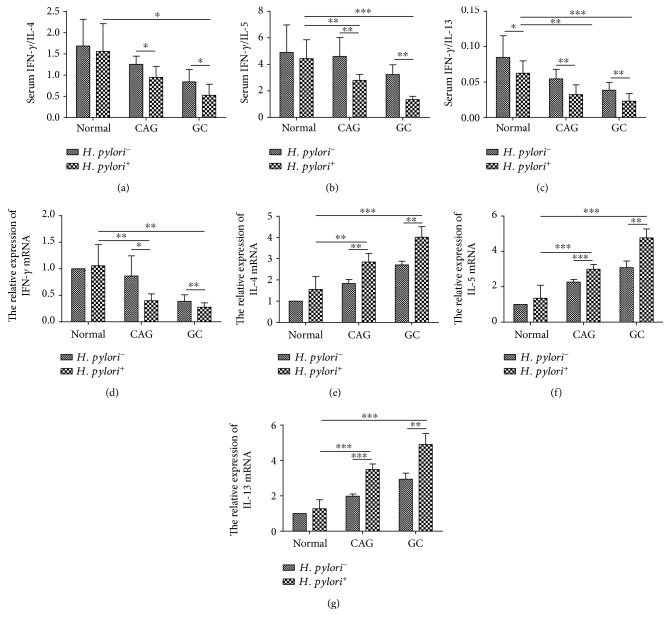
The imbalance of type 1 cytokine/type 2 cytokine expression was found in *H. pylori*-associated gastric diseases. The ratio of plasma IFN-*γ*/IL-4 (a), IFN-*γ*/IL-5 (b), IFN-*γ*/IL-13 (c) decreased along with the progression of gastric cancer, including chronic atrophic gastritis (CAG) and gastric cancer (GC). Moreover, *H. pylori* infection exacerbated this trend. IFN-*γ* (d) mRNA decreased, but IL-4 (e), IL-5 (f), and IL-13 (g) mRNA expression increased in PBMCs derived from the *H. pylori*-infected individuals along the gastric cancer progression, when compared to those without *H. pylori* infection. ^∗^*p* < 0.05, ^∗∗^*p* < 0.01, and ^∗∗∗^*p* < 0.001.

**Figure 2:**
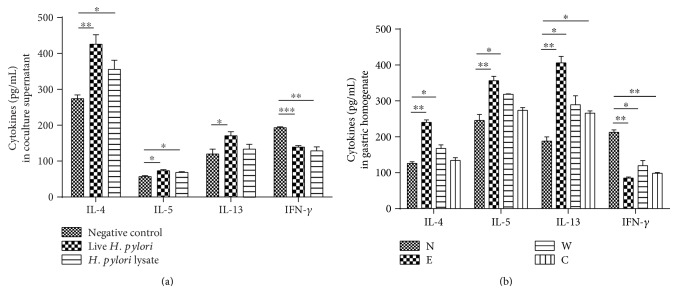
The imbalance of type 1 cytokine/type 2 cytokine expression was also introduced by *H. pylori* infection *in vitro* and *in vivo*. (a) After coculture for 48 hours, the concentration of IL-4, IL-5, and IL-13 in supernatants was increased in the *H. pylori* intervention group, while the expressions of IFN-*γ* reduced. (b) After successful establishment of an *H. pylori-*infected model, the concentration of IL-4, IL-5, and IL-13 was enhanced in gastric homogenate from mice treated with *H. pylori* strains; however, IFN-*γ* in plasma was inhibited, when compared to that in the normal group. Moreover, the East Asian type evoked the most significant change. ^∗^*p* < 0.05, ^∗∗^*p* < 0.01, and ^∗∗∗^*p* < 0.001.

In order to validate this assumption, fresh blood samples were collected from clinical patients and healthy volunteers. Consistent with cytokine microarray, plasma IFN-*γ* concentration decreased in a time course manner in distinct *H. pylori*-involved cancerous checkpoints compared to that of plasmas from normal individuals. The type 2 cytokines, including IL-4, IL-5, and IL-13, were slightly increased but not significantly. However, Th1/Th2 balance was disturbed, when we compared them in a ratio of IFN-*γ*/type 2 cytokines, including IL-4, IL-5, or IL-13, respectively (Figures 1(a), 1(b), and 1(c)). Moreover, as displayed in Figures 1(d), 1(e), 1(f), and 1(g), RT-PCR revealed that IL-4, IL-5, and IL-13 mRNA expressions of PBMC were gradually increased from normal to *H. pylori*^+^ GC-derived PBMCs, which was also significant when compared to their *H. pylori*^−^ counterparts. However, IFN-*γ* mRNA expression was inhibited in the progression of *H. pylori*-associated GC. These results suggested that an impaired type 1 immune response and a potential enhanced type 2 immune response were induced in the progression of *H. pylori*-associated gastric diseases. In order to further confirm this, we have also established an *H. pylori* infection model *in vitro* and *in vivo*. Similarly, we found that type 1 cytokine, IFN-*γ*, was inhibited and type 2 cytokines, like IL-4, IL-5, and IL-13, were augmented or stayed undisturbed in the *H. pylori* infection group *in vitro* and *in vivo* (Figure 2()).

### 3.2. GATA-3-Positive Lymphocytes Were Increased in *H. pylori*-Induced Gastric Diseases

**Figure 3:**
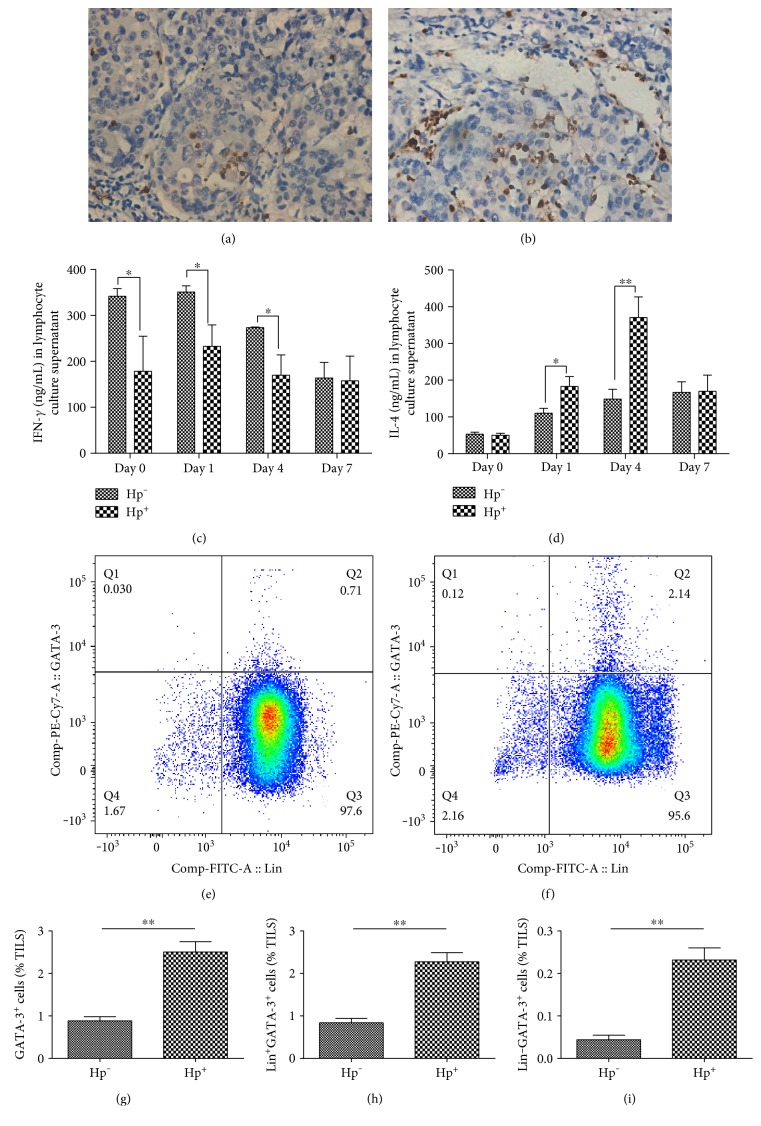
GATA-3 was enhanced in lymphocytes isolated from *H. pylori*^+^ gastric cancer tissues, which was responsible for the skewed type 2 immune response. As shown in the immunohistochemical staining, when compared to the *H. pylori*^−^ gastric cancer tissues (a), GATA-3 expression was significantly upregulated in the interstitial tissues of *H. pylori*^+^ gastric cancer tissues (b). Moreover, the GATA-3^+^ cell indicated a morphological characteristic of lymphocyte. (c, d) Tumor-infiltrating lymphocytes from *H. pylori*^+^ gastric cancer tissues (Hp^+^) showed a stronger secretion of IL-4 but a weaker secretion of IFN-*γ*, when compared to those from *H. pylori*^−^ gastric cancer tissues (Hp^−^). (e–i) After preparation of mononuclear cells from gastric tissues, GATA-3^+^ population was found to increase in *H. pylori*^+^ gastric cancer tissues by flow cytometry. Moreover, Lin^−^GATA-3^+^ population and Lin^+^GATA-3^+^ population were increased in cells from *H. pylori*^+^ gastric cancer tissues, respectively. ^∗^*p* < 0.05, ^∗∗^*p* < 0.01.

GATA-3 has been demonstrated to be involved in the progression of *H. pylori*-associated gastric cancer in our previous findings, which was explained by the decreased Cx32 and Cx43 expression and disordered gap junction *in vitro* and *in vivo* [12]. As a critical transcript factor, GATA-3 was also known to play a decisive role in type 2 immunity, which might partly explain the bias in the chronic *H. pylori* infection. Accordingly, we have detected GATA-3 in gastric cancer tissues and found that GATA-3 was strongly upregulated in the mesenchyme of *H. pylori*^+^ gastric cancer tissues, while the GATA-3-positive cells were nearly much less in the *H. pylori*^−^ gastric cancer tissues or paired normal gastric tissues (Figures 3(a) and 3(b)). More interestingly, lymphocyte populations derived from different gastric cancerous tissues displayed distinct function. After culture *in vitro* for a week, lymphocytes isolated from *H. pylori*-infected gastric cancer tissues (Hp^+^) displayed a vigorous ability to secrete IL-4 and an impaired function of IFN-*γ* secretion, when compared to those isolated from the *H. pylori*-uninfected individuals (Hp^−^) (Figures 3(c) and 3(d)). From the morphological point of view, we inferred that GATA-3^+^ cells were mainly lymphocytes. We have also proved that the increased GATA-3^+^ cells were mainly distributed in the lymphocyte population freshly isolated from the *H. pylori*-infected gastric cancerous tissues (Figures 3(e) and 3(f)). Moreover, overexpression of GATA-3 mRNA was not only found in the PBMCs along the progression of *H. pylori* infection-induced gastric disease but also presented in the *H. pylori*^+^ GC tissues and their derived mononuclear cells, and weaker expression was displayed in the *H. pylori*^−^ gastric cancer tissues or the lymphocytes (Supplementary Figure 1A-B available online at https://doi.org/10.1155/2017/4927964).

### 3.3. Lin^−^GATA-3^+^ Population and Lin^+^GATA-3^+^ Population Were Increased in *H. pylori* Infection

GATA-3, known as a critical transcript factor for both ILC2s and Th2 cells, was found to be increased in *H. pylori*-infected gastric cancer tissues. In order to clarify how the definite population plays a role in *H. pylori*-induced immune response, we tried to identify them by flow cytometry. As the expression of GATA-3 was mainly distributed in Th2 cells, recently, GATA-3 was also found to be highly expressed in ILC2s. Interestingly, we found that Lin^−^GATA-3^+^ and Lin^+^GATA-3^+^ population both increased in the *H. pylori*-infected individuals (Figures 3(e), 3(f), 3(g), 3(h), and 3(i)).

**Figure 4:**
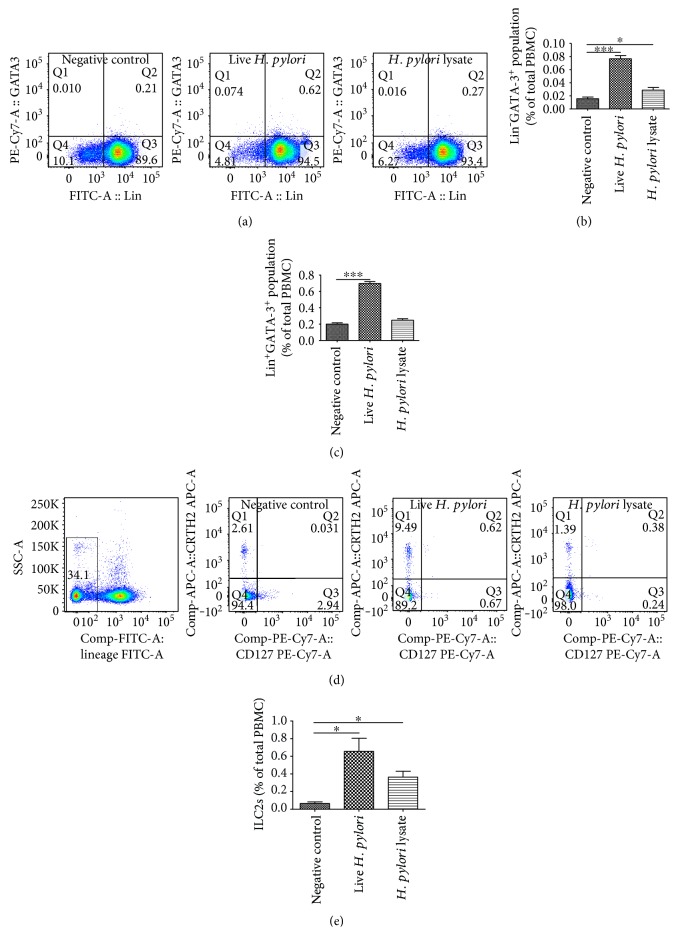
GATA-3^+^ and Lin^−^GATA-3^+^ populations were found to increase after *H. pylori* infection *in vitro*. A coculture system was conducted as described before. (a) After coculture with *H. pylori*-infected GES-1, PBMCs were detected by flow cytometry. GATA-3^+^ population was found to increase after live *H. pylori* infection. (b, c) Moreover, Lin^−^GATA-3^+^ and Lin^+^GATA-3^−^ populations were both found increased. (d, e) ILC2s were also proved to increase after live *H. pylori* infection. ^∗^*p* < 0.05, ^∗∗∗^*p* < 0.001.

To further verify this, a coculture system *in vitro* was conducted as described before [19]. After coculture, PBMCs were detected by flow cytometry; an increase in the percentage of GATA-3-positive cells was found. Moreover, after being cocultured with *H. pylori*-infected GES-1, PBMCs displayed a much higher expression of GATA-3 translationally and transcriptionally (Supplementary Figure 1C-D). Interestingly, there was also a remarkable increase in the Lin^−^GATA-3^+^ and Lin^+^GATA-3^+^ population after coculture (Figures 4(a), 4(b), and 4(c)).

Given that ILC2s are GATA-3-expressing innate lymphocytes and other innate lymphocyte lineages (i.e., ILC1 and ILC3) expressed only weak or little GATA-3. Based on our above findings, it was logical to deduce that ILC2 population in PBMCs was increased after *H. pylori* infection. To verify this, ILC2s, defined as Lin^−^CD127^+^CRTH2^+^, were detected by flow cytometry. As shown in Figures 4(d) and 4(e), ILC2, one of the innate immunity members, was involved in *H. pylori* infection-induced skewed type 2 immune response.

### 3.4. ILC2s Were Found to Be Increased in Clinical *H. pylori*-Infected Individuals

**Figure 5:**
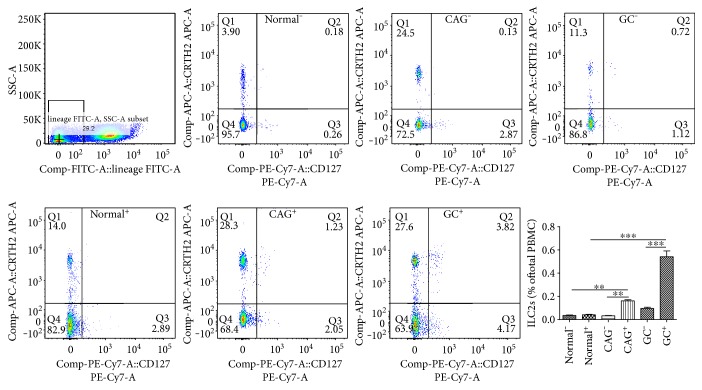
ILC2s, defined as Lin^−^CRTH2^+^CD127^+^, were found to increase in *H. pylori*^+^ chronic gastritis (CAG^+^) and gastric cancer (GC^+^) individuals, when compared to normal individuals. However, there was no difference in the *H. pylori*^−^ counterparts. ^∗∗^*p* < 0.01, ^∗∗∗^*p* < 0.001.

According to the above results, Lin^−^GATA-3^+^ and Lin^+^GATA-3^+^ cells, which indicated a phenotype of ILC2 and Th2, respectively, increased significantly after *H. pylori* infection. As a member of natural innate immunity, ILC2 was demonstrated to play a role in defending microbial pathogens, including virus, parasites, and bacteria. Moreover, ILC2s were an important source of type 2 immune response cytokines and played a vital role in activating and maintaining Th2 immunity. As a result, it was reasonable to deduce that ILC2s were a possible main squeeze for the prevailing type 2 immunity, especially with the enhanced GATA-3 expression. Therefore, ILC2 (Lin^−^CRTH2^+^CD127^+^) count was analyzed by flow cytometry. As expected, ILC2s were found to increase in the *H. pylori* infection group, especially displaying a gradual increase along the disease progression, when compared to the corresponding control group, which was free of *H. pylori* involvement, and healthy volunteers (Figure 5).

### 3.5. An Enhanced GATA-3 Expression and Increased ILC2s Were Also Found with *H. pylori* Infection *In Vivo*

**Figure 6:**
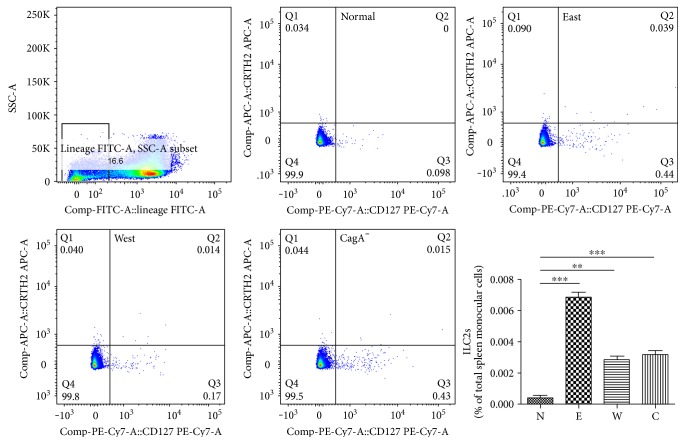
An increased ILC2 (Lin^−^CRTH2^+^CD127^+^) proportion was found *in vivo*. After gavages of different *H. pylori* strains, ILC2 populations in mice were found to increase, especially in the group treated with the East Asian type (E). The Western-type (W) and CagA^−^ (C) *H. pylori* also promoted a slender enhancement of ILC2 at a much lower extent. ^∗∗^*p* < 0.01, ^∗∗∗^*p* < 0.001.

In order to verify the role of GATA-3 and/or ILC2s played in the dominant type 2 immune response induced by *H. pylori*, we have established a series of mouse models by giving gavages of different strains of *H. pylori*. As described above, the enhanced type 2 immune response and inhibited type 1 immune response were also observed in the microenvironment of *H. pylori*-infected C57B/L6 in comparison with that of control groups (Figure 2). ILC2s were also found to be increased in the *H. pylori*-infected group (Figure 6). Moreover, East Asian-type *H. pylori* strain stirred up the most powerful immune response. The mice displayed a distinct pattern of GATA-3 mRNA expression in splenocytes, among them, East Asian-type *H. pylori* was shown to cause the most significant transformation, when compared to the control group given a comparable gavages of PBS (Supplementary Figure 1E).

## 4. Discussion

In a country with high *H. pylori* infection rates, over half of Chinese people are infected with this gram-negative bacilli; however, clinical outcomes vary from different hosts and strains. About 20% of infected individuals develop *H. pylori*-associated gastric disease, while majority remain asymptomatic; immune response caused by reciprocal interaction displays a crucial role in these different outcomes [20, 21]. Abundant evidence demonstrates that the pathogenesis of lesions originated from the infected gastric mucosal epithelium is mostly attributed to the T cell-driven immune responses [3, 8, 22, 23]. As a gastric bacterial pathogen, *H. pylori* causes defensive response immediately upon its adhesion and invasion, “alarmins” will be released from the gastric mucosal epithelium, and lymphocytes are recruited and activated, hence promoting their special cellular immunity and humoral immunity [24].

Both of the recruitment of neutrophils and mast cells contribute critically to the effector phase of *H. pylori* clearance through their effective cytokines [25]. Even so, chronic and persistent infection of *H. pylori* has been proved to be the most frequent clinical status. Plentiful evidence suggests that specific T cell subsets and their distinctive cytokine profiles contribute to the control of *H. pylori* infections on the one hand and to the associated gradual gastric plastic pathology on the other. Generally speaking, upon microorganism infection, *H. pylori* neutrophil activation protein and cytotoxin-associated gene A (CagA) expressed on the bacterial surface will stimulate macrophages, whose activated cytokines are secreted, including IL-12 and IL-23, which are potent stimuli for type 1 immune response [26]. A preferential activation of Th1 response has also been suggested in different animal models in the early phase of *H. pylori* infection, such as mice, monkeys, and dogs experimentally infected with *H. pylori* [27–29]. Simultaneously, macrophages, Treg, or Th2 were activated to relieve the inflammatory activity, especially in *H. pylori* strains carrying the cag pathogenicity island, which confers an increased risk of atrophic lesions and carcinogenesis in Chinese carriers [30–32]. Furthermore, chronic inflammatory and negligent microenvironment of the stomach induced by *H. pylori* is thus usually promoted.

Type 2 immunity which consists of responses dominated by the cardinal type 2 cytokines has been demonstrated to be responsible for *H. pylori*-associated chronic inflammation, especially for the carcinogenesis and immune escape of gastric cancer [33]. According to our results, ILC2s, one of the important sources of type 2 cytokines, might be a novel candidate actor and promoter for preferred type 2 immunity in *H. pylori* infection. Firstly, GATA-3 expression is upregulated by infection of *H. pylori in vitro* and *in vivo*. Secondly, the proportion of ILC2s is significantly increased in the *H. pylori*-infected patients and *H. pylori* infection models *in vitro* and *in vivo*, which accords with the findings of Bie et al. that ILC2 contributed to immunosuppressive microenvironment in the Chinese gastric cancer patients, of which, most are *H. pylori* positive [34]. More importantly, in many situations, ILC2s and Th2 cells collaborate during type 2 immune responses [35–38].

In addition to their established role in innate immunity, ILC2s function to promote Th2 adaptive immunity. Firstly, ILC2s can enhance CD4^+^ T cell proliferation *in vitro* [36]. Secondly, ILC2s can activate and maintain the production of type 2 cytokines from Th2. As an initiator, IL-4 promotes the Th2 differentiation. IL-4 produced initially by ILC2s can activate Th2 cells, initiate, and/or enhance the capacity of the IL-4 production [39]. More importantly, the interaction between ILC2s and CD4^+^ T cells involves the costimulatory molecule OX40L and the cytokine IL-4, which are mainly derived from ILC2s [37]. DCs and/or macrophages also participate in ILC2-induced Th2 differentiation. Moreover, Th cells also contribute to ILC2 activation. MHC class II expressed on ILC2s will activate T cells to produce IL-2, which will act back on ILC2s to produce more IL-4 and activate the Th2 consistently [35, 36, 40]. IL-4 produced by preliminary cells (including ILC2s and developing Th2 cells) may activate Th2 cells in a paracrine and/or autocrine manner in return. Therefore, a regulatory feedback mechanism can be concluded as a fundamental principle for the interaction between ILC2s and Th2 cell differentiation [41]. The two pathways could be concluded as ILC2/IL-4/GATA-3/Th2/IL-4 and CD4^+^T/IL-2/ILC2/IL-4/Th2. However, whether direct ILC2 and T cell interaction is critical for initiating and promoting Th2 cell differentiation or this interaction mainly reflects collaborative relationship of ILC2s and Th2 cells at the effector stage in *H. pylori* infection requires further investigation.

GATA-3 also plays an important role in both Th2 and ILC2 development and interaction. Hoyler et al. have demonstrated that GATA-3, a critical transcription factor for Th2 cells, is also an important regulator for ILC2 lineage specification and maintenance by using excellent genetic and cellular approaches [42]. Mjosberg et al. have proved that GATA-3 is essential for the production of type 2 cytokines (e.g., IL-13, IL-5, and IL-4) by human ILC2s and also depicted the signaling pathways involved in regulating ILC2s' function [43]. Moreover, persistent expression of GATA-3 is important for the maintenance of a mature phenotype and cytokine profiles. Temporal deletion of GATA-3 affects the ILC2s' progenitor pool in the bone marrow and will also inhibit the Th2 differentiation in the peripheral immune system [14]. Though not validated in the present study, it is logical to hypothesize that overexpression of GATA-3 in lymphocytes is a critical molecular mechanism for increased ILC2s and Th2 cells in *H. pylori* infection-associated gastric disease.

In conclusion, our present study showed that there is a type 2 immunity dominant in the development of *H. pylori* infection-associated gastric disease. Activation of ILC2s upon *H. pylori* infection might be responsible for the dominant type 2 immune response. Furthermore, increased expression of GATA-3 induced by *H. pylori* invasion might be a possible mechanism explanation for the skewed type 2 immune responses, and further validation is still warranted.

## Supplementary Material

Supplementary Figure 1. (A) GATA-3 mRNA was found increased in PBMCs derived from the H. pylori infected individuals along the gastric cancer progression, when compared to those without H. pylori infection. (B) GATA−3 mRNA was increased in the H. pylori (+) GC tissues (Hp(+)) and mononuclear cells derived from H. pylori (+) GC tissues, but displayed a weaker expression in the H. pylori (−) gastric cancer tissues (Hp(−)) or its infiltrated lymphocytes. (C) GATA−3 mRNA increased in the PBMCs cocultured with live H. pylori infected or H. pylori lysates stimulated GES−1. (D) Representative western blotting analysis of GATA−3 protein level, which was increased after coculture. (E) GATA−3 mRNA from H. pylori infected mice spleen were also increased, when compared to the mice inoculated with PBS. ＊p<0.05, ＊＊p<0.01, ＊＊＊p< 0.001.

## Figures and Tables

**Table 1:** Gene primer sequences were used for RT-PCR analysis.

	Forward primers (5′–3′)	Reverse primers (3′–5′)
Human IL-4	TCTCACCTCCCAACTGCTTC	TGTCTGTTACGGTCAACTCG
Human IL-5	ATCTTTCAGGGAATAGGCACA	TTGCAGGTAGTCTAGGAATTGGT
Human IL-13	CACTTGCCTTGGCGGCTTTG	CCTTCTGGTTCTGGGTGATG
Human IFN-*γ*	ACTTCTTTGGCTTAATTCTC	AGTTCCATTATCCGCTACAT
Human GATA-3	GGAGTGTGTGAACTGTGGGG	TTCGCTTGGGCTTAATGAGG
Human GAPDH	GAGCCACATCGCTCAGACAC	CATGTAGTTGAGGTCAATGAAG
Mouse GATA-3	GACCCGAAACCGGAAGATGT	TTTTCCACGTACTGCGCG
Mouse *β*-actin	CGTGCGTGACATCAAAGAGAA	AAGCTCGTCCTCTACCGG
