# Supplementary material for: Group 2 Innate Lymphoid Cells Are Involved in Skewed Type 2 Immunity of Gastric Diseases Induced by Helicobacter pylori Infection
Source: Mediators Inflamm. 2017 Sep 12;2017:4927964. doi: 10.1155/2017/4927964 (PMC5613366; doi:10.1155/2017/4927964)
Supplement: Supplementary file 1 — Supplementary Figure 1. (A) GATA-3 mRNA was found increased in PBMCs derived from the H. pylori infected individuals along the gastric cancer progression, when compared to those without H. pylori infection. (B) GATA−3 mRNA was increased in the H. pylori (+) GC tissues (Hp(+)) and mononuclear cells derived from H. pylori (+) GC tissues, but displayed a weaker expression in the H. pylori (−) gastric cancer tissues (Hp(−)) or its infiltrated lymphocytes. (C) GATA−3 mRNA increased in the PBMCs cocultured with live H. pylori infected or H. pylori lysates stimulated GES−1. (D) Representative western blotting analysis of GATA−3 protein level, which was increased after coculture. (E) GATA−3 mRNA from H. pylori infected mice spleen were also increased, when compared to the mice inoculated with PBS. ＊p<0.05, ＊＊p<0.01, ＊＊＊p< 0.001. [file 4927964.f1.doc]

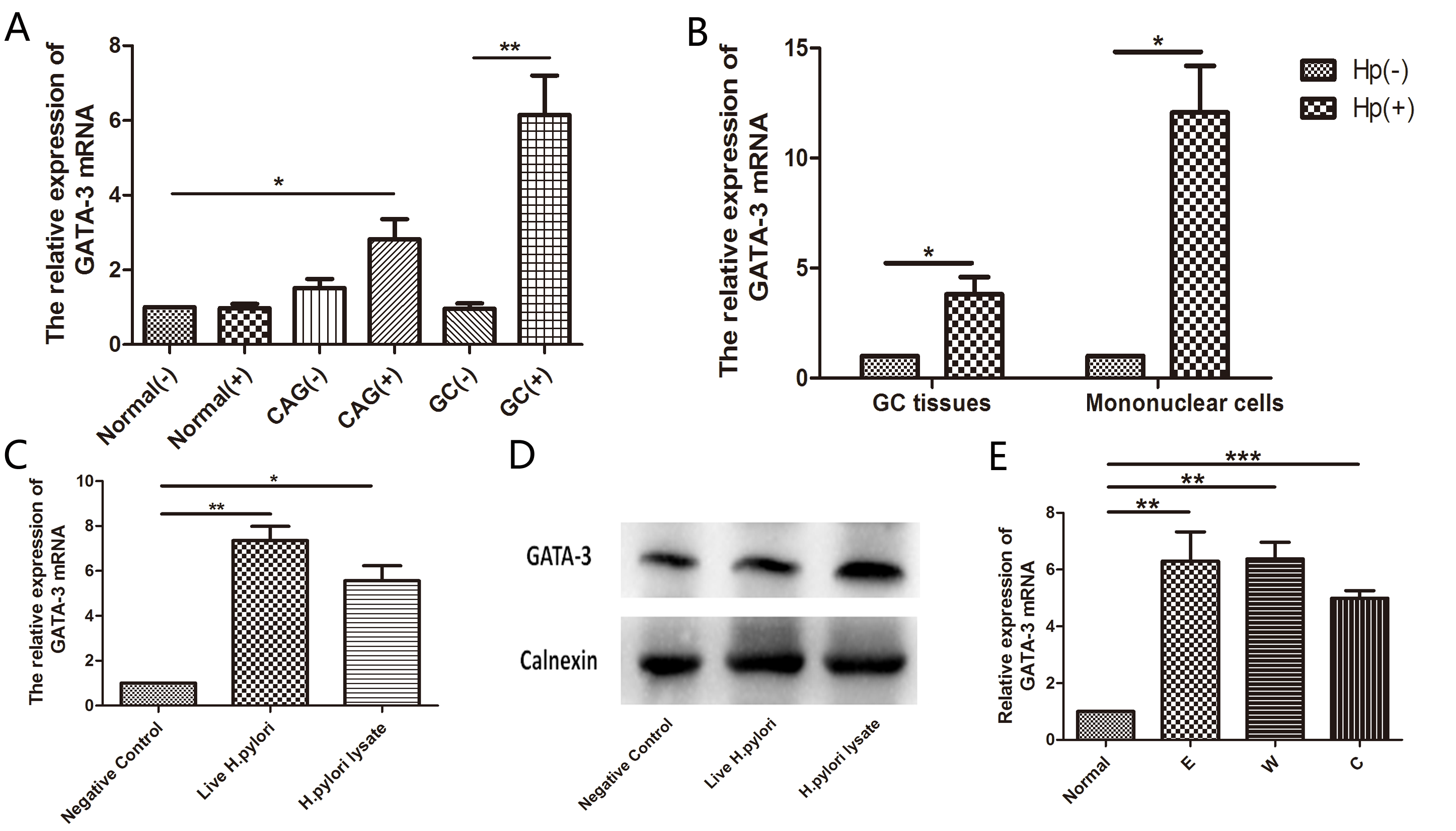
Supplementary Figure 1. (A) GATA-3 mRNA was found increased in PBMCs derived from the *H. pylori* infected individuals along the gastric cancer progression, when compared to those without *H. pylori* infection. (B) GATA-3 mRNA was increased in the *H. pylori* (+) GC tissues (Hp(+)) and mononuclear cells derived from *H. pylori* (+) GC tissues, but displayed a weaker expression in the *H. pylori* (-) gastric cancer tissues (Hp(-)) or its infiltrated lymphocytes. (C) GATA-3 mRNA increased in the PBMCs cocultured with live *H. pylori* infected or *H. pylori* lysates stimulated GES-1. (D) Representative western blotting analysis of GATA-3 protein level, which was increased after coculture. (E) GATA-3 mRNA from *H. pylori* infected mice spleen were also increased, when compared to the mice inoculated with PBS. *, *p* <0.05, **, *p* <0.01, ***, *p* < 0.001.
